# Engineering of a *Bacillus amyloliquefaciens* Strain with High Neutral Protease Producing Capacity and Optimization of Its Fermentation Conditions

**DOI:** 10.1371/journal.pone.0146373

**Published:** 2016-01-11

**Authors:** Hui Wang, Lian Yang, Yanhai Ping, Yingguo Bai, Huiying Luo, Huoqing Huang, Bin Yao

**Affiliations:** Key Laboratory for Feed Biotechnology of the Ministry of Agriculture, Feed Research Institute, Chinese Academy of Agricultural Sciences, Beijing, P. R. China; National Renewable Energy Lab, UNITED STATES

## Abstract

The neutral protease has high potential for industrial applications, and attempts to improve enzyme expression level have important application values. In the present study, a neutral protease-encoding gene, *Banpr*, was cloned from *Bacillus amyloliquefaciens* strain K11, and a genetic manipulation method specific for this difficult-to-transform strain was developed for the high-level expression of neutral protease. The recombinant plasmid pUB110-*Banpr* was constructed in *Bacillus subtilis* strain WB600 and then transformed into strain K11 under optimized conditions. A positive transformant 110N-6 with the highest protease secreting capacity on skim milk plates and great genetic stability for more than 100 generations was selected for further study. Optimization of the fermentation conditions increased the enzyme activity of strain 110N-6 to 8995 ± 250 U/ml in flask culture and 28084 ± 1282 U/ml in 15-l fermentor, which are significantly higher than that of the native strain K11 and industrial strain *B*. *subtilis* AS.1398, respectively. The high expression level and extreme genetic stability make *B*. *amyloliquefaciens* strain 110N-6 more favorable for mass production of neutral protease for industrial uses.

## Introduction

Microbial proteases are among the most important hydrolytic enzymes which dominate the worldwide enzyme market [1]. *Bacillus* species are prolific producers of extracellular proteases with a wide range of applications, particularly in the detergent, food, pharmaceutical, leather and chemical industries [2, 3]. Of the numerous proteases, neutral proteases from *Bacillus* spp. are zinc metalloproteinases with pH optima around 7, and are extensively used in nitrogen control, food industry to reduce the bitterness [4], fresh fish waste treatment [5], soy modification for use as flavors, milk protein modification and in animal feeds preparation [6]. Though extremely important in industry process, the expression level of neutral protease is only one-fifth to one-tenth of other proteases. The low yield results in relatively high price and consequently limits the wide use of neutral protease. Increasing yield would be of great significance in industrial production of neutral protease.

Overexpression of related gene by genetic engineering is an enabling technology for improving enzyme productivity. Since 1983, the first cloning and expression of the neutral protease structural gene from *Bacillus stearothermophilus* CU-21 in *B*. *subtilis* was reported [7, 8], the period of gene modification on neutral protease improvement has begun. Up to now, numerous *Bacillus* neutral proteases have been characterized and genes encoding for these enzymes have been cloned and sequenced from *Bacillus subtilis* [9–11], *Bacillus amyloliquefaciens* [12, 13], *Bacillus cereus* [14], *Bacillus stearothermophilus* [15, 16], *Bacillus thermoproteolyticus* [17, 18], *Bacillus nematocida* [19], *Bacillus caldolyticus* [20], etc. A number of these studies have undertaken expression of neutral proteases by multicopy plasmids or by modifying regulatory elements in heterologous hosts like *Escherichia coli* or *B*. *subtilis*. However, due to the toxicity of protease to heterologous host and slow processing of pro leader peptide, recombinant expression of neutral protease is very difficult and hampers its commercialization. Therefore, it will be highly efficient to express the multiple copies of a neutral protease gene in host strain by utilizing its own expression elements, allowing for appropriate post-translational modification and localization.

*Bacillus subtilis* strain AS.1398, an industrial strain widely used in China, was developed by traditional mutation breeding approaches including UV treatment and chemical mutagenesis and optimization of fermentation parameters and medium over the last century. The yield of neutral protease produced by this strain reached 8000‒10000 U/ml. However, over the past two decades, the neutral protease-producing capacity of strain AS.1398 has not been further improved. Compared with *B*. *subtilis* host, *B*. *amyloliquefaciens* is also extremely important for commercial processes for exoenzymes as they frequently exhibit higher capacity of secreting proteins than *B*. *subtilis*. The high level production of extracellular enzymes makes *B*. *amyloliquefaciens* interesting particularly for an industrial microbiologist [21], but *B*. *amyloliquefaciens* is genetically different from *B*. *subtilis*. The shortage of efficient genetic transformation method has hindered applying recombinant DNA techniques to this organism.

*Bacillus amyloliquefaciens* strain K11 shares extremely high sequence identity with strain 1398 and has high neutral protease-producing ability. However, traditional mutagenesis techniques have failed to improve its yield of neutral protease. In the present study, increased production of neutral protease from this difficult to transform strain was achieved by transforming *B*. *amyloliquefaciens* K11 with recombinant construct pUB110-*Banpr* containing its native promoter, ribosomal binding site, initiation codon and signal sequence to overexpress neutral protease gene. In combination with optimization of fermentation process, the neutral protease yield was increased significantly in both shake flasks and fermentor.

## Materials and Methods

### Strains, plasmids and culture conditions

*Escherichia coli* Trans1-T1 cells (TransGen) were routinely grown in LB medium supplemented with 100 μg/ml ampicillin and used for gene cloning. Details of other bacterial strains and plasmids used in this study are listed in Table 1. *Bacillus* cells were cultured on LB medium at 37°C supplemented with 20−30 μg/ml kanamycin when necessary. To induce neutral protease production, strain K11 was cultured at 37°C with constant agitation of 200 rpm in the seed medium containing (w/v) 1% tryptone, 1% beef extract and 0.5% NaCl. The fermentation medium consisted of (w/v) 4.0% corn meal, 3.0% wheat bran, 3.0% soybean meal, 0.4% Na_2_HPO_4_, and 0.03% KH_2_PO_4_.

**Table 1:** Bacterial strains and plasmids used in this study.

Strains/plasmids	Relevant properties ^a^	Source
*Escherichia coli* Trans1-T1	F-φ80*(lacZ)*Δ*M15*Δ*lacX74hsdR*(*rk-mk+*)Δ*recA13*98*end*A1*ton*A	TransGen
*E*. *coli* EC135/pM. Bam	*E*. *coli* EC135 harboring pM. Bam plasmid	[22]
*Bacillus subtilis* WB600	*nprE aprE epr bpr mpr*::*ble nprB*::*bsr Ʃvpr*	[23]
*Bacillus subtilis* AS.1398	Neutral protease industrial strain	This study
*Bacillus amyloliquefaciens* K11	Wild type strain with high neutral protease-producing capacity	ACCC19735
*Bacillus amyloliquefaciens* 110N-6	*B*. *amyloliquefaciens* K11 harboring *Bacillus* sp. high copy number vector pUB110-*Banpr*	This study
*Bacillus amyloliquefaciens* 110N-N3	*B*. *amyloliquefaciens* K11 harboring pUB110-*Banpr* vector	This study
pEASY-T3	*E*. *coli* cloning vector; Amp^R^	TransGen
pUB110	*Bacillus* spp. expression vector; Kan^R^	BGSC
pUB110-*Banpr*	pUB110 harboring *Banpr* gene of *B*. *amyloliquefaciens* K11	This study

^a^ Amp^R^, ampicillin resistance; Kan^R^, kanamycin resistance

### Molecular identification of strain K11

Genomic DNA of strain K11 was extracted according to the standard protocols using the TIANamp Bacteria DNA Kit (Beijing). PCR amplification of the 16S rRNA and *gyrB* genes were performed using bacterial universal primers 27F/1492R and degenerate primers UP-1S/UP-2Sr (Table 2), respectively. The PCR bands of 1.6 kb and 1.2 kb were purified using Gel extraction kit (Omega) and cloned into the pEASY-T3 vector for sequencing. The sequences were submitted to NCBI website for analysis.

**Table 2:** Primers used in this study.

Primers	Sequences (5′ → 3′)
27F	AGAGTTTGATCCTGGCTCAG
1492R	ACGGCTACCTTGTTACGACTT
UP-1S	GCCTGCATCATCTGGTTTGGGARATHGT
UP-2Sr	CTCTCAGCGGCAGAATNGCYTGRAA
npr-F	CTCTCACTAAACAGCAAGTCAT
npr-R	GGCATCACACCCGGTGTGGAA
P1	GCGGAAAAAAGGAAGGACGGACAGATCAAGAACTGTTATGGCTACAAGATA
P2	AGGCGCCCATTCCAAATGAAAACTGAAGTTGCTCAAAAAAATCTCGGTCAG
P3	CTGACCGAGATTTTTTTGAGCAACTTCAGTTTTCATTTGGAATGGGCGCCT
P4	TATCTTGTAGCCATAACAGTTCTTGATCTGTCCGTCCTTCCTTTTTTCCGC

### Quantitative assay of protease activity

Casein was used as the substrate for protease activity assay and prepared as follows: 1.0 g of casein was dampened with a few drops of 0.5 M NaOH, followed by addition of 80 ml of Na_2_HPO_4_-NaH_2_PO_4_ buffer (6.02 g/l Na_2_HPO_4_∙12H_2_O, 0.5 g/l NaH_2_PO_4_, pH 7.5). The mixture was stirred constantly while boiled in water for 30 min until the casein was dissolved thoroughly. The mixture was then cooled to room temperature, and the pH and final volume were adjusted to 7.5 and 100 ml, respectively. The protease activity was determined by using the Folin-phenol method of the People's Republic of China GB/T 23527–2009. Briefly, 0.5 ml of 1% (w/v) casein solution was preheated at 30°C for 10 min, followed by addition of 0.5 ml of 30°C-preheated appropriately diluted enzyme solution. The reaction mixture was incubated at 30°C for 10 min, and 1 ml of 400 mM trichloroacetic acid (TCA) was added to terminate the reaction. The reactions with enzyme addition after TCA were used as controls. After centrifugation at 13,000 g for 5 min, 1 ml of the supernatant was added into a test tube containing 5 ml of 400 mM Na_2_CO_3_ and 1 ml of Folin-phenol reagent, followed by incubation at 30°C for 20 min. The absorbance was measured at 680 nm. One unit of protease activity was defined as the amount of enzyme that hydrolyzed casein to produce 1 μg of tyrosine per minute.

### Expression vector construction in *B*. *subtilis*

To construct of expression vector in *B*. *subtilis*, POE-PCR method [24] was employed to increase the ligation efficiency of recombinant plasmid. Primer pairs P1/P2 and P3/P4 (Table 2) were used to amplify the vector backbone of pUB110 and *Banpr* gene, respectively. Both PCR products were gel purified and used as templates for the second cycle of PCR without primers. Through natural competence transformation [25], 1−10 μl of the secondary PCR product was transformed into *B*. *subtilis* WB600 competent cells, and Kan-resistant transformants were selected on LB agar plates containing 20 μg/ml kanamycin. Positive transformants were further confirmed by PCR amplification, restriction digest and protease activity detection on 3% (w/v) skim milk plates (forming halos).

### Production of *Ba*NPR in its parent strain

Recombinant plasmid pUB110-*Banpr* was then transformed into its parent strain to achieve overproduction of *Ba*NPR. To introduce foreign DNA into strain K11, a series of parameters, including screening of the optimal growth medium, optimization of electroporation buffers, changing of electroporation conditions, altering the type of DNA transformed (DNA extracted from *E*. *coli* Tran1-T1, *E*. *coli* EC135/pM.Bam and *B*. *subtilis* WB600), amount of DNA added (50−100 ng), were examined to prepare the electro-competent cells of *B*. *amyloliquefaciens* K11 [26–31]. Ultimately, the competent cells of *B*. *amyloliquefaciens* K11 were prepared according to protocol following. Briefly, an overnight LB culture of strain K11 was diluted 100-fold with fresh LB medium and grown until the OD_600_ reached 0.5. Penicillin of different concentrations (0−100 μg/ml) was then added. The cultures were incubated overnight until the OD_600_ values were 20−50% of that of controls without penicillin addition under the same conditions, followed by cooling on ice for 20 min. The cells were collected by centrifugation at 4°C, 8000 g for 5 min, and washed three times with ice-cold electroporation buffer (500 mM sorbitol, 500 mM mannitol, and 10% glycerol). After resuspension in 1 ml of electroporation buffer, 80 μl of competent cells were mixed with 5 μl of plasmid pUB110 or pUB110-*Banpr* (~ 250 ng) extracted from *B*. *subtilis* WB600. The mixture was kept on ice for 5 min, transferred to a pre-chilled 1 mm gap electroporation cuvette, and immediately electroporated via a Bio-Rad Gene Pulser. Right after the pulse delivery, the cells were diluted into 1 ml of LB medium containing 500 mM sorbitol and 380 mM mannitol (LBSM medium). After incubation at 37°C for 3 h with agitation at 50 rpm, the cells were spread on LB plates containing 30 μg/ml kanamycin for selection of positive transformants containing the episomal plasmids. The number of positive transformants was counted after overnight growth at 37°C, and the copy numbers of *Banpr* were verified by PCR and restriction digest. Colonies with larger clearance zones on skim milk plates were picked up and grown in shake flasks.

### Fermentation of recombinant *Ba*NPR in shake flasks

The transformants selected above were cultured at 37°C for 10−12 h with agitation at 200 rpm in seed medium containing 30 μg/ml of kanamycin with the parent strain K11 as a control. Ten milliliters of the seed culture (20% inoculum volume) were inoculated into 500 ml of Erlenmeyer flasks containing 50 ml of fermentation medium and then aerobically grown at 37°C for 2−3 days in a rotary shaker (200 rpm). The culture supernatants were collected at regular intervals and centrifuged at 13,000 g for 10 min for protease activity assay and electrophoresis analysis on SDS-PAGE. The bands of neutral protease were then excised from the gel for protein identification by matrix assisted laser desorption/ionization time of flight (MALDI-TOF) mass spectrometry at institute of apicultural research of Chinese Academy of Agritual Sciences. The transformant exhibiting the highest enzymatic activity was selected for optimization of culture conditions.

### Optimization of fermentation conditions for *Ba*NPR production

Extracellular protease production is greatly influenced by physical factors such as pH, temperature and incubation time and by others factors such as media composition and presence of metal ions [32]. The main fermentation factors, including temperature (30 to 37°C), initial pH (6.0 to 8.0), loading volume of culture medium (50 to 200 ml) and inoculum size (2 to 20%), were optimized for maximum production of *Ba*NPR. Moreover, the effect of amino acids (0.05% of methionine, lysine and histidine) and metal ions (0.1% or 0.2% of Ca^2+^, Mg^2+^ and Zn^2+^) on *Ba*NPR production were also tested.

### Genetic stability of recombinant strain of *Ba*NPR

Continuous passage cultivation was conducted to monitor the segregation stability of recombinant plasmid pUB110-*Banpr* in *B*. *amyloliquefaciens* K11. The recombinant strains were streaked onto the seed medium agar plates supplemented with 30 μg/ml kanamycin and incubated at 37°C for 12 h. One colony was picked and cultivated in the liquid seed medium containing 30 μg/ml kanamycin for 12 h. The culture was then diluted 10^4^-fold to 10^6^-fold and plated on seed medium agar plates containing antibiotic for growth of 14−16 h. The colony numbers in each diluted-fold plates were counted, and one hundred colonies were picked randomly and dotted on seed medium agar plates with and without 30 μg/ml kanamycin simultaneously. The number of colonies on each plate was counted and compared to determine the frequency of plasmid loss. The dilution process was repeated at 10-generation intervals for 10 times.

Enzyme digestion of the recombinant plasmids was conducted to determine the structural stability of pUB110-*Banpr* in *B*. *amyloliquefaciens* 110N-6. The plasmid extracted from the 100 generations (plasmid B) and the plasmid constructed originally (plasmid A) were digested with multiple restriction enzymes (*Bgl*II/*Nco*I, *EcoR*I and *Bgl*II/*Eco*RV) to confirm the presence of the recombinant plasmid and to examine its structural stability. Plasmid B was then re-transformed into *B*. *amyloliquefaciens* K11 to detect the production of neutral protease. The plasmid from the newly constructed strain *B*. *amyloliquefaciens* 110N-N3 (plasmid C) was extracted and digested with the same enzymes above.

### Large-scale fermentation of *Ba*NPR in fermentor

Enhanced neutral protease production by the engineered *B*. *amyloliquefaciens* strain was scaled up from flask to 15-l bioreactor (Bioengineering) containing 10-l of fermentation medium under batch fermentation conditions with industrial strain *B*. *subtilis* AS.1398 as controls. The fermentation medium was sterilized *in situ* at 121°C for 20 min and inoculated with 300 ml [3.0% (v/v)] of incubated seed culture under OD_600_ = 0.4−0.6. The agitation speed was set to 750 rpm and compressed air was sparged at a flow rate of 1.0 vvm. The initial pH of the fermentation medium was adjusted to 7.0 with NaOH and was monitored but not controlled during fermentation. The fermentor temperature was controlled by the following thermal gradient program: started and maintained at 32°C for initial 3 hours; increased at a rate of 1°C/h until 40°C; decrease at a rate of 1.5°C/h until 32°C; isotherm at 32°C until the end of fermentation. Samples were collected at variable intervals, and the neutral protease activity was estimated. Fermentation was performed in three fermentors simultaneously, and the fermentation procedures were repeated for three times.

### Nucleotide sequence accession numbers

The nucleotide sequences for the 16s rRNA, *gyrB* gene and neutral protease gene (*Banpr*) of *B*. *amyloliquefaciens* K11 were deposited in the GenBank database under accession number KM603513, KM603514 and KM603515, respectively.

## Results and Discussion

### Identification of neutral protease high producing strain

Phylogenetic analysis of the 16s rDNA gene of strain K11 against known sequences indicated that strain K11 is affiliated with the genus *Bacillus*. *gyrB* encodes the subunit B protein of DNA gyrase and has been proved to be a better molecular marker than the 16S rDNA gene for the study of phylogenetic and taxonomic relationships at specie levels of *Bacilus* [33]. The *gyrB* gene of strain K11 had the highest identity of 99% to that of *B*. *amyloliquefaciens* Y2, thus strain K11 was termed as *B*. *amyloliquefaciens*. It was deposited at Agricultural Culture Collection of China under registration No. ACCC19735.

### Cloning and sequence analysis of *Banpr* from *B*. *amyloliquefaciens* K11

By using the primer set npr-F/npr-R (Table 2), a gene fragment of 2533 bp was amplified from strain K11, including a 519-bp upstream fragment, a 1563-bp open reading frame (ORF) of *Banpr* and a 448-bp downstream fragment. A possible terminator sequence was found immediately downstream of the ORF in the 3′-non-coding region. Compared with the corresponding gene sequence of strain Y2, *Banpr* had 12 nucleotide substitutions: 2 occurred in the promoter region, 2 in terminator region, and 8 in the ORF region.

**Fig 1:**
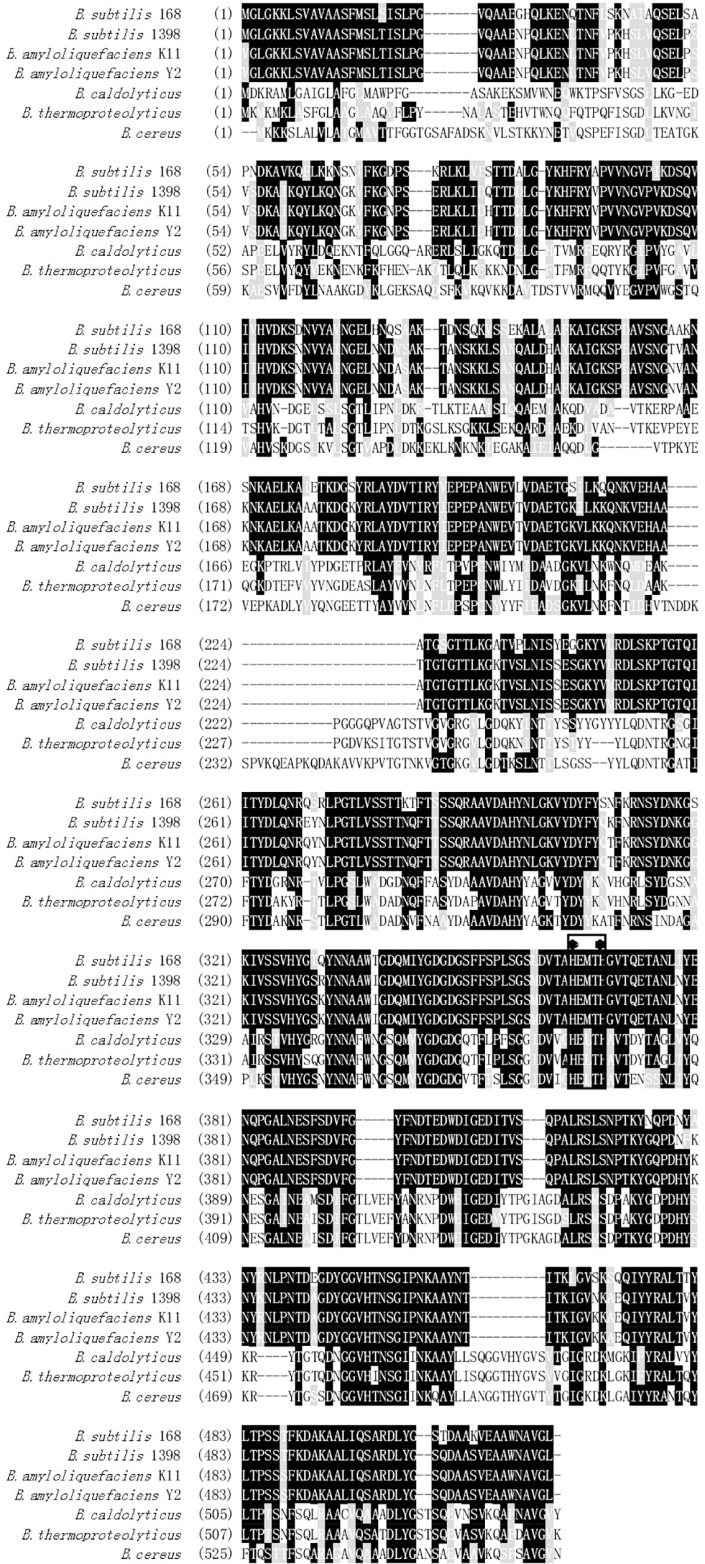
Multiple amino acid sequence alignment of NPR. Amino acid sequence alignment of *Ba*NPR from *Bacillus amyloliquefaciens* K11 with the NPR from *Bacillus amyloliquefaciens* Y2, NPRE from *Bacillus amyloliquefaciens* (*Bacillus velezensis*) (P06832), NPRE from *Bacillus subtilis* 168 (P68736), NPRE from *Bacillus cereus* (P05806), THER from *Bacillus thermoproteolyticus* (P00800), and NPRE from *Bacillus caldolyticus* (P23384) using the ClustalW program. Two histidine residues in active site are indicated by asterisks.

Multiple alignment of the deduced amino acid sequence of *Banpr* with other neutral proteases is shown in Fig 1. Deduced *Ba*NPR exhibited 100%, 96%, and 83% sequence identity with the neutral proteases from *B*. *amyloliquefaciens* Y2, *B*. *subtilis* AS.1398, and *B*. *subtilis* strain 168 [11], respectively. It consists of a signal peptide of 27 residues, a pro-region of 194 residues, and a structural region of 300 residues. The calculated molecular mass was predicted to be 32.7 kDa. Modeled *Ba*NPR shows the typical structure of metalloproteinase: the pro-peptide, the catalytic domain, and the haemopexin-like C-terminal domain. Two histidine residues (His 143 and His 147) and catalytic glutamate residue (Glu 144) in the zinc-binding motif sequence and one glutamate residue (Glu 167) chelating the active-site Zn^2+^ were also identified in the putative tertiary structure.

### Heterologous expression in *B*. *subtilis*

Due to the low transformation efficiency using *Bacillus* as the expression host, many shuttle plasmids have been developed to carry out the initial cloning steps in *E*. *coli* and then the recombinant plasmids were transferred into *Bacillus* strains. To facilitate vector construction, we initially attempted to construct the shuttle expression vector of *Banpr* in *E*. *coli*. Only a few ligation mixtures of *Banpr* and linearized shuttle vectors were successfully transformed into *E*. *coli* Tran1-T1 competent cells as shown on screening plates, and nonsense mutations existed in all colonies. Similar phenomenon also occurred in the report of Tran *et al*. [9]. Wang *et al*. [34] had ascribed the lytic effect of *nprE* gene product on *E*. *coli* cells and removed the ribosome binding site (RBS) of *nprE* to achieve expression and secretion. Therefore, we constructed a recombinant plasmid harboring its original promoter, SD sequence, coding sequence and terminator sequence of *Banpr* in *B*. *subtilis* WB600, instead of in *E*. *coli*. These elements are very efficient for expression of *Bacillus* genes that are toxic to *E*. *coli*.

By using the POE-PCR method introduced by You et al. [24], recombinant plasmid pUB110-*Banpr* was successfully constructed in *B*. *subtilis* WB600. The positive transformants were verified by PCR amplification, and the accuracy was confirmed by enzyme digest and sequencing. The colonies harboring pUB110-*Banpr* showed clear halos on skim milk plates, while the controls (protease-deficient *B*. *subtilis* WB600 carrying empty plasmid pUB110) did not. It revealed that *Banpr* with its original expression elements was functionally expressed in *B*. *subtilis* WB600.

### Transformation of recombinant plasmid pUB110-*Banpr* into *B*. *amyloliquefaciens* K11

It is quite difficult to incorporate foreign DNA into *Bacillus* cells due to the lack of efficient gene transformation system and the existence of R-M systems [22]. Optimization of hypertonic agents, pulse voltage, and electroporation buffers have been reported to improve the transformation efficiencies; however, these protocols are highly species or strain specific, and the efficiency is relatively variable among strains even using the same protocol [30]. *B*. *amyloliquefaciens* has much thicker cell wall. Modifying the cultivation conditions, growth conditions, or amending electroporation parameters such as applied voltage and capacitance and the quality of DNA had no effect on transformation of strain K11. Zhang et al. [30] reported that the walls of vegetative cells grown in semi-complex NCM medium are relatively loose and easy to form pore during electroporation, and consequently more accessible by exogenous plasmid. However, *B*. *amyloliquefaciens* K11 showed no growth in NCM medium. Cell-wall-weakening agents gly and DL-thr of *B*. *amyloliquefaciens* TA208 [30] did not improve the transformation efficiency in *B*. *amyloliquefaciens* K11, either. The reason might be that the cell-wall carbohydrates varied in the glycosyl compositions of strains TA208 and K11 or different chemical dosages were used. Strain K11 was obtained with the ampicillin concentration up to 100 μg/ml, which is much higher than the previously reported level [30, 31]. The different mode of restriction-modification between *B*. *subtilis* and *E*. *coli* makes the transformation of recombinant plasmid extracted from *B*. *subilis* WB600 much easier into strain K11 than those from *E*. *coli*. Recombinant plasmid pUB110-*Banpr* was extracted from *B*. *subtilis* WB600 and transformed into *B*. *amyloliquefaciens* K11 via optimized electroporation method. The positive transformants acquired kanmycin-resistant characteristic were selected on LB plates containing 30 μg/ml of kanamycin and subsequently verified by PCR amplification and enzyme digest.

### Overproduction of *Ba*NPR in *B*. *amyloliquefaciens* K11

Five transformants harboring pUB110-*Banpr* showed apparent transparent zones on skim milk plates and were further cultivated in shake flasks. Great variance was detected on the protease-producing ability of transformants and parental strain K11 (Table 3). The transformant 110N-6 showed distinguished neutral protease producing capacity (7460 ± 51 U/ml) over the parental strain (2694 ± 49 U/ml). Because vector pUB110 is an autonomously replicating plasmid, the different levels of protease production should be attributed to the various damages occurred during the electroporation process. The increase of protease productivity in transformant 110N-6 is likely ascribed to the increased copy numbers of *Banpr* and high transcriptional levels.

**Table 3:** Production of recombinant *Ba*NPR by *Bacillus amyloliquefaciens* harboring pUB110-*Banpr* in shaker flasks^a^.

Transformants harboring pUB110-*Banpr*	Enzyme activity (U/ml)
110N-4	5747 ± 186
110N-6	7460 ± 51
110N-7	3427 ± 172
110N-11	5287 ± 133
110N-16	6891 ± 270
K11	2694 ± 49

^a^ All data are shown as mean ± SD (n = 3)

**Fig 2:**
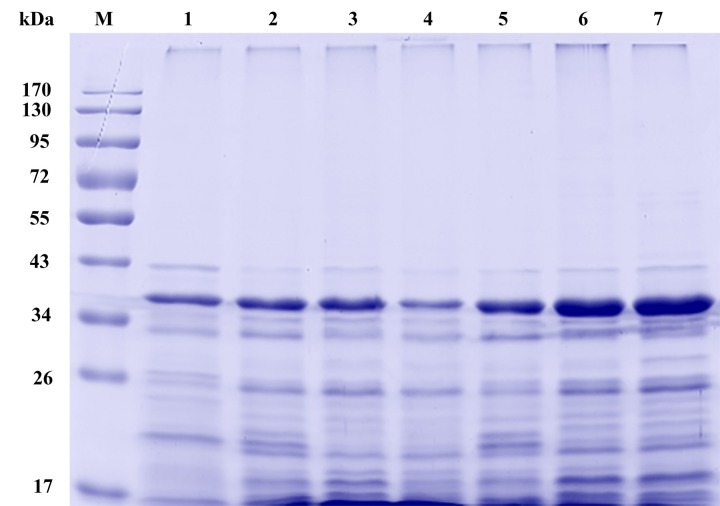
SDS-PAGE analysis of recombinant *Ba*NPR in *Bacillus amyloliquefaciens* K11. Lane: M, the molecular mass standards; 1–3 and 5–7, the different transformants harboring pUB110-*Banpr*; 4, the transformant harboring the empty vector pUB110.

A protein band with a molecular mass of about 37 kDa was observed in the culture supernatants of all strains as shown in SDS-PAGE (Fig 2). The apparent molecular weight of *Ba*NPR was a little larger than its calculated value (32.7 kDa). Further MALDI-TOF analysis of the band verified the identity of *Ba*NPR and revealed complete incision of the signal peptide (S1 Fig). The slow mobility of expressed protein on the SDS gel also has been reported in other neutral and alkaline proteases [35, 36]. The similar phenomenon might be ascribed to certain modifications of the expressed protein in host cells.

### Genetic stability of recombinant plasmid

As shown in Table 4, the recombinant plasmid pUB110-*Banpr* in *B*. *amyloliquefaciens* 110N-6 kept constant during the serial passage process. One hundred colonies were randomly picked up from generations up to 100, and no clone lost in the media with or without antibiotic (S2 Fig). The restriction patterns of plasmid DNA were identical as shown in S3 Fig, suggesting that neither rearrangement nor deletion occurred, and nor obvious spontaneous mutation of DNA sequence generated during the long sequential replication in transformant 110N-6. The neutral protease activity of the newly constructed *B*. *amyloliqefaciens* 110N-N3 at generation 100 reached 7983 U/ml at 72 h in flasks, further confirmed the stability of the plasmid. All the results above proved that the hereditary stability of pUB110-*Banpr* in *B*. *amyloliquefaciens* 110N-6 was excellent for industrial application. Because it is impractical to add antibotic in large-scale fermentation, plasmid stability is an important factor for industrial applications without selective pressure.

**Table 4:** Segregation stability of pUB110-*Banpr* in recombinant *Bacillus amyloliquefaciens* 110N-6 by continuous passage cultivation^a^.

No. of generation	The number of colonies in 10^−5^-diluted plates	The number of colonies in 10^−6^-diluted plates
1	370 ± 2	54 ± 1
10	400 ± 6	50 ± 4
20	320 ± 1	48 ± 2
30	410 ± 5	58 ± 5
40	380 ± 3	49 ± 1
50	340 ± 1	43 ± 2
60	330 ± 4	47 ± 1
70	310 ± 2	56 ± 4
80	290 ± 3	45 ± 3
90	280 ± 6	48 ± 3
100	260 ± 3	43 ± 1

^a^ All data are shown as mean ± SD (n = 3)

There are two possible mechanisms to underlie plasmid stability, i.e. segregation stability and structural stability. Under normal circumstances, the structural stability of a plasmid can be achieved by the rigorous selection of host strains. Segregation instability accounts for the majority of plasmid instability. Due to the variations of host’s metabolic load and copy numbers caused by defective partitioning, cells without plasmid are produced. Adverse environmental conditions and growth competition from the cells without plasmid may further promote the instability of the plasmid. The copy number of the recombinant plasmid represents an important factor to affect plasmid stability. When the cell divides, the plasmids are randomly assigned to the daughter cells. The higher copy number the plasmid has, the lower probability the plasmid-free cells and the more stable the plasmid.

In this study, the growth curves of recombinant strain 110N-6 and parent strain K11 showed no difference (data not shown), and two recombinant plasmids pUB110-*Banpr* and pKan300-*Banpr* were constructed simultaneously. Recombinant *B*. *amyloliquefaciens* F20 harboring pKan300-*Banpr* was genetically instable under the same conditions as recombinant *B*. *amyloliquefaciens* 110N-6 containing pKan300-*Banpr*, and lost all recombinant plasmid pKan300-*Banpr* after 8 generations (data not shown). The only difference between these two plasmids is that the *E*. *coli* expression elements existed in the pKan300-*Banpr* vector of *B*. *amyloliquefaciens* F20. Therefore we speculate that it is the *E*. *coli* expression elements that cause plasmid instability in *Bacillus* hosts. Therefore, *B*. *amyloliquefaciens* 110N-6 with distinguished genetic stability was selected for further optimization and large-scale fermentation.

### Optimization of the culture conditions for *Ba*NPR production

**Fig 3:**
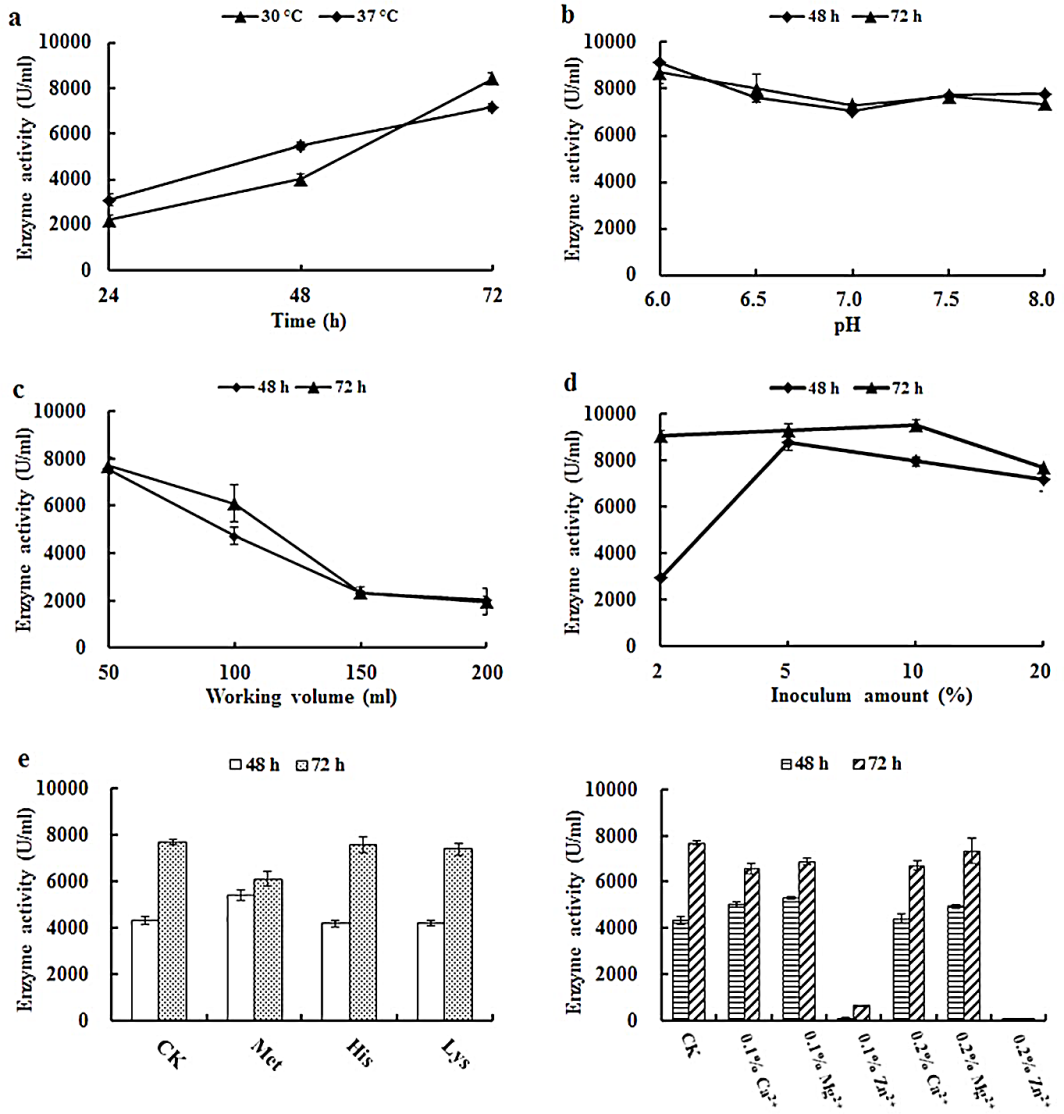
Optimization of culture conditions for *Ba*NPR production in *Bacillus amyloliqufaciens* 110N-6. a Effect of temperature on enzyme production. b Effect of pH on enzyme production. c Effect of working volume on enzyme production. d Effect of inoculum size on enzyme production. e Effect of different amino acids on enzyme production. f Effect of different metal ions on enzyme production. Each value in the panel represents the means ± SD (n = 3).

**Fig 4:**
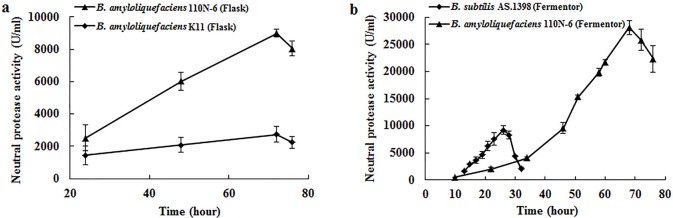
Comparison of the production of neutral protease in shake flasks and fermentors. a The enzyme activity of r-*Ba*NPR and *Ba*NPR produced by *Bacillus amyloliquefaciens* 110N-6 and *Bacillus amyloliquefaciens* K11 on flask level under optimized conditions. b The enzyme activity of r-*Ba*NPR and *Bs*NPR produced by *B*. *amyloliquefaciens* 110N-6 and *Bacillus subtilis* 1398 on large-scale batch production in the fermentor. Each value in the panel represents the means ± SD (n = 3).

The optimal conditions for *Ba*NPR production by transformant 110N-6 were investigated in 500 ml shake flasks. As shown in Fig 3, the optimal fermentation conditions of 110N-6 were determined as initial pH at 6.0, 30°C, 200 rpm/min, 60−72 h, 50 ml of working volume, and 5% inoculum capacity (Fig 3A−3D). Under the optimized conditions, the yield of r-*Ba*NPR increased significantly to 8995 ± 250 U/ml, while that of *Ba*NPR only reached 2739 ± 472 U/ml (Fig 4A). Some amino acids are known to induce protease production. Thus the effect of methionine, lysine and histidine on *Ba*NPR production was also tested. Addition of lysine and histidine had no effect on *Ba*NPR production, but methionine enhanced the production at 48 h (Fig 3E). Moreover, the enzyme production was slightly inhibited by Ca^2+^ and Mg^2+^, and completely suppressed by Zn^2+^ (Fig 3F).

### Large-scale fermentation of *Ba*NPR

*B*. *subtilis* AS.1398 has been used for production of neutral protease for several decades in industries. To justify whether the engineered strain 110N-6 with high activity and excellent genetic stability was contestable for industrial production of neutral protease, both recombinant strain *B*. *amyloliquefaciens* 110N-6 and industrial strain *B*. *subtilis* AS.1398 were subjected to large-scale fermentation. As shown in Fig 4B, the neutral protease production by *B*. *subtilis* AS.1398 and *B*. *amyloliquefaciens* 110N-6 reached the maximum of 9218 ± 754 U/ml and 28084 ± 1282 U/ml at about 26 h and 68 h, respectively, and declined after that. Although the rapid production of neutral protease by *B*. *subtilis* AS.1398 is desirable, combined analysis of the yield and fermentation duration reveals that *B*. *amyloliquefaciens* 110N-6 is more efficient (413 U/ml·h vs. 355 U/ml·h) in the production of neutral protease. Therefore, *B*. *amyloliquefaciens* 110N-6 constructed in this study with high yield of neutral protease and excellent genetic stability will overcome the bottlenecks (low yield and high price) of neutral protease production, and has the potential to replace *B*. *subtilis* AS.1398 as the industrial strain for neutral protease production.

## Conclusions

In conclusion, a neutral protease-encoding gene, *Banpr*, was cloned from *B*. *amyloliquefaciens* K11 and engineered for overexpression in its native host. By optimization of the fermentation conditions, the *Ba*NPR yield of engineered strain K11 harboring the *Bacillus* expression vector pUB110-*Banpr* reached 28084 U/ml in a 15-l fermentor, which is much higher than that of the widely used industrial strain *B*. *subtilis* AS.1398. Moreover, *B*. *amyloliquefaciens* 110N-6 showed excellent genetic stability. All these properties indicate r-*Ba*NPR represents a valuable neutral protease candidate for economic mass-production and industrial applications.

## Supporting Information

S1 Fig Peptides of *Ba*NPR identified by MALDI-TOF mass spectrometry. (EPS)

S2 Fig Colonies of *Bacillus amyloliquefaciens* strain 110N-6 after subcultivation of 100 generations. a Seed medium agar plate without kanamycin. b Seed medium agar plate with 30 μg/ml kanamycin.
(EPS)

S3 Fig Enzyme digestion profile of plasmid pUB110-*Banpr*. Lanes: M, the molecular mass standards; 1–3, plasmid A (the originally constructed plasmid) digested with enzymes *Bgl*II/*Nco*I, *EcoR*I and *Bgl*II/*EcoR*V, respectively; 4–6, plasmid B (the plasmid extracted from *Bacillus amyloliquefaciens* 110N-6) digested with the same enzymes; 7–9, plasmid C (the plasmid extracted from *Bacillus amyloliquefaciens* 110N-N3) digested with the same enzymes.
(EPS)
